# The crafting of hope: Contextualising add-ons in the treatment trajectories of IVF patients

**DOI:** 10.1016/j.socscimed.2021.114317

**Published:** 2021-10

**Authors:** Manuela Perrotta, Josie Hamper

**Affiliations:** Department of People and Organisations, School of Business and Management, Queen Mary University of London, UK

**Keywords:** IVF, Add-on treatment, Hope, Treatment decision-making, United Kingdom

## Abstract

The proliferation and popularity of additional treatments in IVF, also known as add-ons, has generated widespread discussion and controversy in the UK, where concerns have addressed the lack of evidence to support the efficacy and safety of these treatments, their cost, and their connection to a wider context of privatisation of fertility treatment. Drawing on 42 interviews with IVF patients, this article explores the role of hope in the appeal of add-ons from the patient perspective. The analysis is presented in two parts: firstly, we investigate the role of hope in patients’ decision-making on treatment, contextualising add-ons in the broader trajectory of their IVF experience; secondly, we examine how patients navigate the offer of add-ons, focusing on the role of hope in how they rationalise their decisions on whether to include them in their fertility treatment. Our analysis shows how patients craft their hope to navigate the increasing number of available options in their quest to find the treatment(s) that will “work” for them. We suggest that the imperative for patients to explore all options is intensified with the emergence of add-ons, which produces a novel context and version of a “hope technology”.

## Highlights

• The role of hope in the appeal of IVF add-ons.
• Patient perspectives on the inclusion of add-ons in fertility treatments.
• Contextualisation of add-ons in patients' IVF trajectory.
• In-depth analysis of how IVF hope narratives shape treatment decisions of IVF patients.
• How patients craft hope in relation to add-ons.

## 1 Introduction

In the last few decades, In-Vitro Fertilisation (IVF) has become widespread worldwide. Although IVF birth rates have increased steadily over the years, success rates remain low overall: according to data published by the Human Fertilisation and Embryology Authority (HFEA), in the UK the average birth rate per embryo transferred was 23% in 2018 (HFEA, 2020). Low success rates in the context of a highly-privatised sector (in England in 2018 only 35% of IVF treatments were NHS-funded) have fostered the proliferation of additional treatments, or “add-ons” as they are popularly defined in the public debate, that aim to increase the chances of success. Although these treatments have been heavily criticised for lacking robust evidence of their efficacy (Heneghan et al., 2016), they are widely available and in recent years, over 70% of UK patients have included add-ons in their fertility treatment (HFEA, 2019a).

In the UK, this controversy has received extended media coverage, including a BBC Panorama documentary (Inside Britain's Fertility Business, 2016) and regular articles in newspapers criticising IVF clinics for taking financial advantage of desperate couples (see for instance, Tompkins, 2019). This debate has had three focus points: the spread of add-ons as an effect of IVF privatisation, their costs, and the lack of evidence supporting them. These concerns have been echoed by the scientific literature. Scholars suggest that the commercialisation of add-ons is part of a wider speculative turn in IVF, with the sector attracting an increasing amount of venture capital and private equity investment (Van de Wiel, 2019, 2020, 2020). Studies reviewing how add-ons are offered by British IVF clinics (Spencer et al., 2016; Van de Wiel et al., 2020) criticise the lack of clarity regarding their safety and effectiveness. For these reasons, some IVF professionals are critical toward the uncontrolled spread of add-ons (Rutherford, 2017; Repping, 2019) and advocate caution in offering potentially non-beneficial add-ons to vulnerable patients without solid evidence (Harper et al., 2017).

Although the debate has focused on commercialisation and the additional costs charged by private clinics without solid evidence to justify the use of add-ons, some add-ons are widely used in both public and private clinics. Some private clinics do not charge for add-ons that are included in their standard packages. NHS clinics also often include some of the most established add-ons in their all-inclusive treatment (Perrotta and Geampana, 2020), which can be either publicly or privately funded at costs that are similar to basic packages offered by some private clinics. The inclusion of add-ons in these cases is considered less controversial as patients are not charged extra for them, yet considering the lack of public funding in this sector, some consider it unethical for the NHS to invest in unproven treatments.

The potential profiteering from unproven add-ons has been framed in the media coverage as “Selling Hope” (Wilkinson, 2019), pushing vulnerable couples to attempt, and often request, any available additional treatment in order to increase their chances of having a baby. The role of hope has been analysed extensively in influential works on IVF in the social sciences (Franklin, 1997; Becker, 2000), which express concerns about the unique IVF appeal of offering hope for a biological child to people who could not cultivate hope otherwise. Despite the, often heated, debate on add-ons, the extended literature on IVF has overlooked how couples make decisions regarding the inclusion of add-ons in their fertility treatments. Drawing on 42 interviews with IVF patients, this article fills this gap, examining the role of hope in add-ons’ demand. Focusing on an analysis of patients' perspectives, this article does not aim to investigate what Debora Spar (2006) defines “the baby business”. However, Spar's argument is relevant to contextualise our findings, as it shows how in many contemporary reproductive global markets (such as IVF, eggs/sperm, and surrogacy) the product on offer is “simultaneously hope and medicine” (2006, p. 35).

## 2 The paradoxes of hope in medicine

Being, in general, positively connoted within medicine, hope is considered central to developing strategies that can promote health (Little and Sayers, 2004; Eliott and Olver, 2007). In medical sociology and anthropology, hope has often been conceptualised as a resource that can help individuals persist through obstacles and (re)act in the face of hardship, as for instance with chronic illness (Mattingly, 2010). However, the paradoxical nature of hope (Mattingly, 2010; Alaszewski and Wilkinson, 2015; Brown et al., 2015) has also been underlined. For instance, Mattingly argues that hope “involves the practice of creating, or trying to create, lives worth living even in the midst of suffering, even with no happy ending in sight” (2010, p. 6). Hope is required to act against and adapt to current circumstances, but cultivating hope can involve the risk of anticipating unrealistic futures that make patients more vulnerable to despair.

In recent years, the risks of hope have been increasingly recognised in the social science literature on health and medicine. Scholars have argued that hope can be excessive (Brown, 2003), unrealistic (Nabi and Prestin, 2016), or even false (Rettig et al., 2007) and that it has the power to propel unproven treatments that have no benefit or are even harmful for patients (Petersen et al., 2014). The downsides of hope have been explored in articulations of “the political economy of hope” (Del Vecchio Good et al., 1990; Novas, 2006; Petersen, 2015), which aim to underline how individual and personal endeavours are framed in a collective understanding of hope for a better future, which is based on biomedical promise. The desire of patients to participate in clinical trials or unproven and expensive treatments is fostered by biopharmaceutical companies and healthcare systems that have financial interests in selling their products. In Western biomedicine, narratives of hope are dominant (Lupton, 2003) and part and parcel of the great promise of science and technological innovations (Mulkay, 1993; Brown and Michael, 2003). On the one hand, hope has been used by bio-industries to mobilise resources and legitimise new forms of clinical intervention (Brown, 2005). On the other, the increasing availability of novel treatments and therapies offers new options and raises patients' expectations in terms of possible available futures (Ezzy, 2000; Kaufman, 2015). Although we recognise that this dynamic of hope is shaped by the social context in which hope is enacted, this article focusses on what Brown and colleagues (2015) term the “lived experiences of hoping”, to stress the role of hope in sustaining patients’ perseverance against uncertainty and coping with their vulnerability. Recent research in the context of other emerging reproductive technologies shows that hope narratives have implications for patients, as they shape not only patients experience but also their actions (Herbrand and Dimond, 2018). Thus, the aim of this article is to explore how hope narratives influence patient decision-making on whether to include add-ons in their fertility treatment.

## 3 IVF as a hope technology

In a pioneering study exploring the experience of the first generation of IVF users in the UK, Franklin defines IVF as a “hope technology” (1997) to stress that IVF is able to offer hope to people who struggle with infertility “as much if not even more than a ‘successful’ outcome, which leads it to be seen as a desirable option, even when it is expected to fail” (1997, p. 310). Although the IVF success rate is statistically low (and was even lower at the time of Franklin's research), it remains significantly higher than not intervening – as often patients approach IVF after years of involuntarily childlessness. Similarly, earlier Danish research (Koch, 1990) had shown how the traditional feminist critique on IVF, based on its risks and its dubious capacity to produce a child, was not shared by infertile women, who were following a different rationality based on a desire for a biological child. Franklin (1997) reinforces this argument showing how IVF patients are often motivated by the desire for a reproductive resolution (i.e., to find resolution in having tried and to accept their inability to have biological children), regardless of the actual outcome of treatments.

In her work on American couples undergoing IVF treatments, Becker (2000) notes that patients are unable to consider alternatives to a biological child (such as adoption or living childless) until they have exhausted all their medical and financial resources. Becker's interviewees present their IVF experience as having simultaneously too many options (in terms of treatment) and no other options (outside of IVF). Both Franklin (1997) and Becker (2000) claim that patients' persistence against failures and their desire to “try anything possible” is often a tool to prevent future regret and obtain a reproductive closure. Becker (2000) suggests that patients' need “to do something” about their infertility reflects American notions of individualism and responsibility for health, while the cultural imperative to use available technology against adversity pushes individuals to accept and embrace the experimental nature of IVF. Similarly, Franklin (1997) argues that IVF patients “living in hope” for a baby represent a more general Western belief in scientific progress and technological embodiment. Both scholars discuss the twofold nature of hope: it is necessary to “keep going” through IVF to see it through, but it also entails an ongoing belief in its potential that is difficult to desist. Becker (2000) underlines that patients need to temper hope against the limitless nature of IVF, in which each new cycle offers new chances of a pregnancy at high emotional and financial costs. Franklin (1997) argues that the flipside of hope is that it can be disabling, when medical assistance becomes a dependency: “coming close to pregnancy, or achieving a ‘chemical pregnancy’, or even simply viewing her own ‘fertility’ through scans, can make it harder for a woman to accept her infertility than it might have been beforehand” (1997, p. 292).

A more recent social science literature has investigated non-medical egg freezing as a technology invested with a particularly future oriented hope for a genetically related child. Unlike the hope narratives of IVF patients, where hope is focused on the more immediate treatment success (a pregnancy) or reproductive resolution, egg freezing allows women to preserve and prolong hope (Van de Wiel, 2015), thus “banking time” to negotiate future fertility (Waldby, 2010). Participants in a study by Baldwin (2019) described multiple narratives of hope including, importantly, the hope that their frozen eggs would not be needed.

Drawing on the rich literature on hope presented above, in this article we analyse the role of hope in patients’ experience of IVF, focusing on how they decided whether to include any add-ons in their treatment. Despite the heated debate on add-ons, research on the perspective of patients is currently lacking. The aim of this article is not to take sides in the debate on the availability of add-ons, their costs, or the evidence supporting them. Rather, it aims to close a gap in the literature through an analysis of how patients navigate available add-ons and how narratives of hope shape their decisions.

## 4 Methodology

The data presented in this article emerge from a larger study that considered professional and patient perspectives on technological innovations in IVF (Perrotta and Geampana, 2020). This paper focuses specifically on findings from interviews with 42 patient participants, including 34 women going through IVF, seven male partners and one female partner. All our participants had at least one of the most common add-ons included in one of their treatments. As the current definition of add-on is porous and there is not a consensus as to what constitutes an add-on and what does not, we opted for an inclusive approach that acknowledged all the treatments our interviewees referred to as add-ons. To cover the complexity of treatment options and capture the experience of patients navigating both the public and private treatment contexts, participants were recruited for interviews through two routes. Between May and October 2018, 22 participants were recruited by research nurses at collaborating NHS fertility clinics, all of which offered at least one of the add-ons under scrutiny by the HFEA (2019a) to their patients. These interviews took place in person at the clinics and primarily involved patients undertaking fertility treatment that was publicly funded, although this was not always the case and some patients were entirely self-funded or paying for additional tests or treatments.

A second approach to recruitment took place between November 2019 and March 2020 via an online survey, resulting in an additional 20 interview participants. The survey covered a range of topics relating to fertility treatment and included the option for respondents to leave their contact information in order to be contacted for further research participation. This survey was distributed online via our project blog and Fertility Network UK's social media outlets, as well as by previous participants in closed IVF patient support groups. Using survey responses (n = 314) enabled us to more purposefully select participants who had varied experiences of seeking fertility treatment, including their evaluations about add-ons specifically, both via NHS and privately funded avenues. 15 of these were interviewed over the phone, four were interviewed in their homes, and one was interviewed in a café setting.

Interviews were semi-structured, including a broad introductory question that invited the participant to narrate a timeline of events relating to their fertility treatment, followed by questions organised broadly around treatment choices and options, including their approach to add-ons. Interviews varied in length, from 30 to 80 min, giving a total of 34 h of interview recording. A short questionnaire was provided after the interview to collect basic personal information such as age, treatment financing and number of completed embryo transfers. As argued in research from other areas of prenatal healthcare (Farrell et al., 2019), we understand the individual undergoing IVF and their partner (where applicable) as “two members of a decision-making dyad” (ibid. p. 212) and we recognise that patients and partners may have different experiences of fertility treatment options. To explore these potential divergences, the research was designed to interview couples separately, although this was not a strict requirement for participation and one couple was interviewed together at their own request.

In all cases, patients and partners were provided with an information letter, offered the opportunity to ask questions about the research and a week to consider participation prior to signing a consent form, which included consent to record the conversation. Each participant was given a £20 voucher after the interview. The study received ethics approval from the Health Research Authority and local approval from each participating fertility clinic.

At the time of the interview, 16 participants were undergoing or about to start IVF treatment, 16 were pregnant or had pregnant partners, and 10 had one or more child(ren) from previous IVF. Although some of our participants had reached their final cycles, none of them had ended their treatment without being pregnant or having a baby. This is significant in contextualising our analysis of hope in this article as all our participants were still undergoing treatment or reflecting on their experience of IVF after they had attained the hoped-for outcome of a pregnancy or baby. It is also worth noting in relation to add-ons specifically, that approximately half of the participant group did not express any evaluation on add-ons, either because they did not consider them due to financial constraints or because they preferred to follow the advice of their consultant. In this article, we focus on the participants who made active choices or felt the desire to shape the direction of their treatment by evaluating, sometimes critically, the available add-ons in relation to their own treatment and in some cases actively seeking them.

Patients were aged between 29 and 41, with a mean age of 35, and partners were aged between 33 and 47, with a mean age of 38. We experienced difficulty in recruiting men for interviews, which echoes a well-documented tendency in research on reproduction (Culley et al., 2013). Moreover, while the aim was not to recruit a demographically diverse participant group, the heterogeneity of our sample, which was largely white, middle class and heterosexual, constitutes a limitation of this research, yet it potentially reflects the significantly lower uptake of fertility treatments by individuals from black and ethnic minority backgrounds (HFEA, 2019b) as well as other sociocultural and structural barriers to accessing fertility treatment in the UK.

Interview recordings were professionally transcribed verbatim. The transcripts were organised and coded in NVivo, and analysed following the basic tenets of a grounded theory approach (Charmaz and Belgrave, 2012). The analysis process involved a first-stage allocation of quotes to largely descriptive codes, followed by a second iteration of themes that attended to how patients articulated their experiences in relation to broader discursive references (Muncie, 2006), such as what constitutes possibility or regret in experiences of IVF. As patients’ narratives of hope emerged as central in how our participants describe taking decisions regarding the inclusion of add-ons in their fertility treatments, our analysis focuses on how hope can mobilise patient agency (Herbrand and Dimond, 2018) and how it shapes their experience and decision making (Brown et al., 2015).

## 5 Findings

Our analysis is presented here in two parts. In the first section, we analyse the role of hope in patients’ experiences, focusing on how hope is essential for them to keep going with fertility treatment, while simultaneously highlighting how they positioned and considered add-ons in relation to the broader trajectory of their IVF experience, which included past procedures as well as expectations, plans and hopes for the future. The second section discusses how patients navigate the offer of add-ons and rationalise their decisions to include or omit these in their fertility treatment with specific reference to their association with the privatisation of fertility treatment in the UK, their cost and their lack of supporting evidence. In the following sections, we quote some participants at length to locate add-ons within their broader experiences of being an IVF patient.

### 5.1 Contextualising add-ons in patients’ IVF trajectory

As established through our review of the literature, hope is central to how fertility patients maintain the motivation to “keep going” and continue treatment in the context of adversity. Patients’ references to hope were complex, indicating how their sense of hopefulness fluctuated – emerging, fading and being actively crafted or curtailed – at different points in treatment. While the ultimate hope of fertility patients was to have a baby, their sense of hope could also have a more immediate focus, involving the hope of making progress through diagnostic or treatment milestones (Franklin, 1997). In this section, we develop an analysis of hope specifically in relation to a contemporary dynamics of evaluating, choosing or declining add-ons. Participants in this study were generally highly reflective of the possibility that they were pursuing or paying for hope through their decisions to include treatment add-ons, where the appeal of add-ons was embedded in a future-oriented responsibility of having exhausted all available options that might improve their chances of pregnancy. Here, we focus specifically on how add-ons enter into a wider fertility treatment trajectory, which could include lengthy periods of diagnostic examinations as well as unsuccessful procedures. We argue that considering add-ons as part of this trajectory helps to understand how patients encounter them in a novel version of the IVF hope narrative, not only in terms of being additional options to a treatment that they already decided to pursue, but also in terms of accepting additional financial and emotional costs, on top of many other costs they had already accepted to incur.

While many participants did not make active choices or feel the desire to shape the direction of their treatment, explaining that they trusted and wanted to follow the advice of their consultant, others described the strongly felt imperative to evaluate or actively seek add-on treatments. For example, one participant described how she was willing to “do anything” and that “it doesn't matter if it hurts, if it takes longer, [or] if it takes more appointments”. Participants often used the language of hope to articulate the problematic sides of making decisions in IVF, explaining that hope can lead to emotionally driven decision-making. The “glimmer of light” referred to in the following quote represents a vision of potentiality that has the power to guide the choices made. This participant refers specifically to her decision to include intralipid infusions in her IVF, which she refers to in the following quote as “the egg yolk thing”, and she also highlights the informal networks of knowledge sharing about add-ons in IVF, including the sharing of experiences between patients:

Before we were put into our treatment plan, I had another friend who had had unsuccessful IVF treatment but she had had this egg yolk thing. And when she told me about it the first thing I did was look it up and I asked about, I asked [my consultant] about it. So I would say that had there been other things that were available to us or, and it was relevant to our treatment, I probably would have [requested them]. Because when … Yeah, like I said it's such an emotionally, you know, you can get into some very dark places and whatever offers a glimmer of light you are drawn towards, I think, yeah. (Patient, became pregnant from first embryo transfer and had baby, awaiting further IVF).

Hope was often something that participants in this research actively pursued following bad news, such as in response to an infertility diagnosis or a negative pregnancy test result after embryo transfer. In these cases, “getting hope back” was coupled with renewing belief in treatment and feeling emotionally ready to continue. Participants often described a sense of pride and achievement at reaching the stage of having one or more viable embryo(s) to transfer, and some talked about the transfer as the closest they had ever been, or may ever be, to a pregnancy. Crucially, having embryos to transfer is not a given in the context of IVF and several participants had been through rounds of egg collection and fertilisation without having any viable embryos to transfer. The following participant reflected on how she had felt positive and hopeful at getting to the stage of embryo transfer, which, even if it did not lead to a pregnancy, would hopefully provide some information that would be valuable in moving forward in treatment:

From when it [the embryo] was transferred, it was like I suddenly got loads of hope. I just thought “oh my God, this could actually work now.” And yeah, it was a really strange time because I'm really negative and I was suddenly really, really positive about it and just couldn't believe it and I said to my husband: until potentially we lose this baby I'm actually pregnant now and I've never been pregnant. So I thought at the very least, you know, at least if the baby sticks for a while then we can really get some answers about what's wrong and at least then, you know, maybe it might work next time if there's something else that I need. (Patient, six years of diagnostic examinations and reducing BMI to meet IVF criteria, pregnant from first embryo transfer).

In a context where there are a vast number of potentially available fertility treatments or procedures, the end of the previous quote underlines the future-oriented notion that “something else” might offer the right treatment that would make a difference for the success of the procedure. In other words, the availability of add-on treatments expands the range of possibility and treatments to try. The following participant articulated the promise of hope that is attached to add-ons specifically, and she located this dynamic within a broader view of IVF as “a bit of a scientific experiment”. Challenging the media portrayal of fertility patients, this participant specified that patients, in her view, do not act in “blind faith” but are making decisions about add-ons in a context where there are many unknowns about the efficacy of treatments and, in the context of privately funded IVF, the possibility of paying for hope has strong appeal:

The way it's portrayed sometimes is that people, because they're in a vulnerable state, they get, they get over-sold certain things because they get sort of sold a bit of a pipedream that if you have this extra or this extra, then that's the thing that will make it work, that, and that these can sometimes add up for people. Which I think sometimes in the media comes from a place of not understanding what it’s like, fertility treatment, and the fact that you're already paying ten grand, it doesn't matter, you know, and you'll do anything and you know, the whole thing is a bit of scientific experiment in that you're just, you know, you, that you have sort of, it's sort of not blind faith but your hope is such that you, if someone says something will work then you might tend to go for it […] You're just paying a lot of money for hope and it, you know, and the add-on *is that*, isn't it? (Patient, had one viable embryo from each egg collection, two embryos transferred over two years, awaiting third egg collection).

Some participants reflected on the timing of their considerations around the inclusion of add-ons in their IVF. In cases where the causes of infertility were not known, many patients described a preference for trying “standard” or “basic” IVF first before pursuing additional treatments. The following participant, however, described how she had chosen to undergo a biopsy for endometrial natural killer cell testing as well as acupuncture from the start, and she was having pre-implantation genetic testing (previously known as pre-implantation genetic screening or PGS) of the embryos created from her next egg collection. For this patient, including and paying for add-ons from early in the treatment process was an attempt to reduce the time to pregnancy and thus reduce the emotional and physical costs of IVF:

We've probably done things earlier than most people would, so probably most people would get to the end of their NHS funded cycles and then go and have all of those tests whereas we decided to have them done earlier on. I guess we're a bit impatient […] We didn't do it [PGS] with our first cycle because you wouldn't, [our consultant] has said it, obviously that you learn something from every cycle and every transfer that you go through, to sort of tailor things and try and make it better for the next time and one of the things that obviously we didn't do for our first was PGS. We had a long think about it and actually if we, if we go down the route of having them all checked then if there are, you know, or if we have six embryos generated and we have four that are aneuploid then that saves us four transfers, the heartbreak of four failed transfers which actually is, it's more a matter of heartbreak, it's a lot of upset so. And also, the hormones that I'm putting in my body, which if I don't have to do that it’s probably better for me in the long run. (Patient, four embryo transfers, preparing for second egg collection).

Participants’ considerations and decisions about add-ons must be positioned as part of a broader experience and trajectory of IVF treatment, which potentially involves significant emotional, physical and financial investment over an extended temporal scale. As demonstrated through the previous quote in particular, add-ons are embroiled in an imperative to “tailor” treatment and “make it better for the next time”, which enables add-ons to always offer something new to try.

### 5.2 Evaluating add-ons: privatisation, cost and evidence

In this section, we focus on the three most controversial aspects of add-ons as represented in the public debate about IVF in the UK: the spread of add-ons as an effect of the privatisation of the field; their costs; and their lack of supporting evidence. Although add-ons emerged in the narratives of our participants as central to their pursuit of treatment success, they often challenged the assumption that fertility patients uncritically accept additional tests or treatments that are offered to them. Patients do not “blindly” accept add-ons but they are working within a treatment context that calls for them to consider all relevant options and weigh these up against overall costs of treatment, which was especially pertinent for those who had pursued IVF through privately-funded avenues. The following participant, who for a short period had considered both a private and an NHS clinic for self-funded treatment, reflected on the main differences they encountered between these clinics:

We had a look at one private clinic before we started this process. Just basically to see what is it, is there something there that is different. Because it doesn't strike us as being logical, why would you go to a private clinic? I understand that you have to pay here [NHS clinic], that's a whole different story. But kind of thing, why would you then go to an outside body if the price is going to be, looking at it online the price seemed to be about the same so we thought we'll go and have a look and see what is it that is different. We did and it wasn't disastrous, it was reasonably clean and reasonably nice but it offered absolutely nothing better than here and in fact, there were quite a few things that were offered that were very dodgy. So we've done our research in terms of all the added extras, all the various scratches and various vitamin supplements, these kind of things and most of them, [according to] the HFEA [have] absolutely zero evidence that they provide any benefit, yet in the private clinic this was something that they were talking about. We'll give you all this special yogurt and everything, all of which are, it would appear, clinically speaking nonsense. (Partner, three embryo transfers over one year, one early miscarriage, awaiting fourth egg collection).

This participant shared concerns about the privatisation of the sector by describing the potential profiteering from unproven add-ons by rapacious private clinics and challenging the additional treatments offered by the clinic they visited, which he deemed to be “very dodgy” and “clinically speaking nonsense”. Specifically, he referred to the endometrial scratch and intravenously administered intralipid infusions (“special yogurt”), neither of which are currently supported by conclusive evidence that they improve pregnancy rates. It is also worth noting that various additional treatments were mentioned in this interview, including things like vitamins, which are not considered add-ons by the medical literature and HFEA. Although the term “add-on” usually refers to additional drugs, tests or lab equipment, the proliferation of products and services offered, from alternative medicine to the wellness industry, adds a further layer of complexity to patients’ navigation of treatment options. In addition, the divide between the NHS (publicly funded) and private fertility clinics was often unclear. In reflecting on their joint decision to request the endometrial scratch and embryo glue in their paid IVF treatment at an NHS clinic, the following partner described the “strangeness” of making choices about whether or not to include certain add-ons:

They're kind of strange choices to be making but then also potentially very beneficial to making things happen but it's quite … I don't know if it's part of being in this country where you kind of [receive] NHS care and it is what it is, and you go and you talk through your choices rather than … It felt a little bit more like kind of picking additional add-ons that you wanted which is not something that I was used to and not as a bad thing but it, again that took quite a bit of getting used to, thinking about, you know, “oh, shall we have this as well” and … Yeah, it's quite a strange mentality to get your head round […] I think with some of the additional things it, you could just keep going and keep going and I think it had all got a bit, we kind of reached that point, that limit point. (Partner, wife had baby from first embryo transfer).

The inclusion of some add-ons in NHS-funded IVF legitimises their use beyond the NHS, where receiving a certain treatment as an NHS patient creates expectations as well as the conditions to request the same treatment as a private patient, even if this is offered at an additional cost. The following participant portrayed a highly individualised sense of responsibility in her decision-making that was tied to a felt imperative to avoid future regret, and she described her process of rationalising add-ons in relation to the high cost of IVF as a whole, where an additional cost of £500 is contextualised against £5000 for the entire procedure:

If I was a private patient and I was offered [a treatment] as an add-on I would have paid for it if I was told it worked. I think, I think you'd pay for anything really. If you're told it works and it's £500 you'd think well, I don't want to not spend the £500 and it didn't work because of that […] I've never felt under pressure to pay for anything but I'm an NHS patient so maybe if I was sitting … See if I have a third cycle, which I hopefully don't want to, but if I have a third cycle that will be as a private patient. So maybe my experience will be different but then, am I going to turn down the [endometrial] scratch when I've had it for all the other ones? And maybe I think well, it didn't work, but maybe I think well, that was other issues that didn't work and actually if I don't then have the scratch we will have paid for all this but … I think you sometimes think that if you're paying all that money you don't want to scrimp and waste £5,000 by not paying for the scratch. (Female patient, five embryo transfers, awaiting further diagnostic examinations and treatment).

Yet participants in this study demonstrated a variety of perspectives on the considerable cost of many add-ons. The first quote below situates costs against possible improvements to chances of pregnancy, however tenuous or minute, and a future-oriented imperative to exhaust all available options, whereas the second presents a more sceptical perspective on the need to invest additional financial resources to obtain a pregnancy:

And then [our consultant] told us about [available add-ons] and because we are in the fortunate position we could pay for it we kind of, just were like yeah, yeah, yeah, we'll do it all, you know, we just do whatever might help. (Female patient, became pregnant from second embryo transfer four years ago and had baby, entered treatment again since, pregnant from second frozen embryo transfer).

If you've got the money to have all of these expensive add-ons that may or may not work great but we don't so I was happy to just go straight down and get on with the treatment and that will be it. And like I said, if they'd offered me the scratch at an extra £200 I probably wouldn't have done it because you know, people get pregnant without having it anyway so, no. (Female patient, three years of diagnostic examinations and reducing BMI to meet IVF criteria, pregnant from first embryo transfer).

In explaining her approach to add-ons, another participant described contrasting modes of rationality between what she terms her “scientific brain” and her sense of “woolly thinking”, where the latter was attached to an intense desire to improve her chances of having a baby despite knowing that some add-ons lack rigorous evidence to support their use:

When I was given the option of things [such as add-ons] my scientific brain then sort of went out of the window and was pushed to one side. Because I was like, well, I don't really care now if there's not any good evidence for this, if it gives me a better chance of having a baby then we'll do it. So I kind of have my standards and then I'm like, you know, I sort of threw them out of the window really. I suppose for me, I kind of have a feeling if some, if something will be actively bad for me if you like … then I wouldn't go down that route […] [or] if it was like, an add-on or a treatment that I'd not really heard of being used on a wide basis or I couldn't see any sort of rationale for it. But I feel that I did go rather woolly and sort of not very scientific thinking towards the end. Because you do, you do start to think well, let's just throw everything at it and see if it works. (Female patient, had one viable embryo from each egg collection, two embryos transferred over two years, awaiting third egg collection).

This participant expressed a desire to “throw everything at it” but also noted three particular reservations about add-on treatments, including whether the treatment in question could cause harm, whether it is widely available, and what constitutes the rationale behind its use. She was familiar with medical terminology around evidence base and previously in the interview she had cited the importance of randomised control trials in supporting new treatments. She also echoed many other participants who expressed a sense of being burdened by the responsibility of navigating the treatment options that are available to them, and it was particularly in relation to weighing up the options that she described the difficulty of maintaining a grasp on her “standards” of evaluating evidence. Throwing “everything at it” aligns with our previous observation that patients present the IVF experience as a quest to find the one treatment or combination of treatments that will “work” for them. Evaluating add-ons is thus connected to patients’ broader acknowledgement of the many unknowns and uncertainties associated with IVF, and these uncertainties are further compounded by the fact that many participants did not have a diagnosis for their infertility beyond it being “unexplained”.

## 6 Discussion

Both the public debate and scientific literature on the add-ons controversy in IVF focus on criticisms regarding the proliferation of add-ons, their high cost, and the fact that none of these treatments have solid evidence of their safety and efficacy at increasing pregnancy rates. As add-ons are very popular in the UK, these criticisms generally imply that patients are vulnerable and irrational in their treatment decision-making regarding add-ons. By contextualising add-ons in patients' IVF trajectory, our analysis offers a more nuanced understanding of how patients make decisions regarding the inclusion of add-ons in their fertility treatments. We use the term “IVF trajectory” to underline the common path, presented in the literature on IVF (Franklin, 1997; Becker, 2000) and confirmed by our participants, that the IVF experience entails. Dealing with infertility means accepting medical support, facing the emotional and financial hardship of IVF treatment, accepting its “experimental nature”, low success rates and that, therefore, multiple attempts need to be made. Our focus on what we term “the crafting of hope” underlines how patients’ decisions about whether to include add-ons are shaped by the dominant discourse of hope in IVF, while at the same time, patients have to continuously curtail and balance hope against other considerations, such as potential harm, emotional costs, and financial resources.

Our findings confirm the dominant IVF discourse on hope illustrated by the literature, which considers hope as essential to persevering against adversity, and show how this discourse is amplified by the proliferation of add-ons, which emerge as novel versions of the “hope technology” (Franklin, 1997). To “keep trying” and “try everything” to avoid future regret are part of the dominant discourse of hope circulating among patients. Our findings show that this discourse is extended to add-ons, which become part of the search for the “right” treatment that will lead to success (a pregnancy) or to a resolution (accepting that is not possible to have a biological child). Similarly to what has been noticed in the case of egg freezing (Waldby, 2010; Van de Wiel, 2015; Baldwin, 2019), add-ons prolong hope in the sense that they foster a narrative according to which the “right” treatment has not yet been found. As the literature has shown, patients' persistence despite failures is closely tied to a reluctance to consider alternatives to a biological child until they have exhausted all their medical and financial resources (Franklin, 1997; Becker, 2000). The proliferation of add-ons offers virtually infinite options to create the illusion that more things can be tried, raising patients’ expectations in terms of possible available futures (Ezzy, 2000; Kaufman, 2015). Our findings confirm that many patients feel the need to “try anything possible” to prevent future regret and obtain reproductive closure, and this influences how they make decisions regarding the inclusion of add-ons in their fertility treatments.

Although our findings show patients' concerns regarding the role of their emotions in their decision making, our participants do not accept add-ons uncritically. Mirroring the literature (Koch, 1990), our findings reveal that patients are not irrational in their decision-making, but they follow a rationality that is based on a desire for a biological child and considers their available options. For instance, when discussing add-ons, patients are concerned about what they actually offer, their costs and the lack of scientific evidence. However, their rationality remains based on achieving the desired outcome (i.e., having a baby) as soon as possible. Patients acknowledge that this rationality does not conform to what is considered a “scientific” one, but in their experience, other issues are relevant in evaluating add-ons. Some participants were concerned about being offered “dodgy things” by private clinics, and they problematised the divide between public and private sectors as represented in the public debate. In terms of the additional costs of add-ons, our participants contextualised these in terms of their personal circumstances (whether they are in the position to afford to invest financial resources in the treatment or not determine their actual options) and in their IVF trajectory (where the cost of additional treatments is usually fractional compared to the cost of the IVF). Similarly, patients’ evaluations of the evidence to support the safety and efficacy of add-ons do not follow a “scientific” rationality, but rather evidence are evaluated on the basis of different criteria, such as whether an add-on is widely available. These criteria are not irrational, but they emerge from the narratives of hope described above, on the basis of which patients seek to find the one treatment or combination of treatments that will “work” for them.

## 7 Conclusion

Despite the often heated debate on add-ons, the extended literature on IVF has overlooked how patients make decisions regarding the inclusion of add-ons in their fertility treatments. This article fills this gap by examining patients' perspectives and focusing on how dominant IVF hope narratives shape patients’ decision-making. Attending to the role of hope is, we argue, central to understanding how add-ons become part of an increasingly complex medical and technological landscape of treatment options for IVF patients. Notably, as we did not interview individuals who decided to end treatment without a baby, our findings cannot offer any insights into how patients manage hope in this situation. Further research is needed to reveal these dynamics.

Our findings confirm the following key elements of the dominant IVF hope discourse (Franklin, 1997; Becker, 2000): the need to “try anything possible” to find reproductive resolution and prevent future regret; the need to accept the experimental nature of IVF; and the need to exhaust all available medical and financial resources before considering alternatives to a biologically related baby. These narratives are fuelled by the availability of so many add-ons, which expands the number of options to try. This can increase the risk of anticipating unrealistic futures (Del Vecchio Good et al., 1990; Brown, 2003, 2005, 2005; Novas, 2006; Petersen, 2015) and make patients more vulnerable to the allure of the IVF market. These narratives encourage the promise of novel (and often unproven) biomedical interventions (Petersen et al., 2014), making it difficult for patients to resist or refuse to consider additional treatments. Our findings contribute to this body of literature, showing how macro level discourses and processes of commercialisation in the offering of new treatments shape not only the possibilities imagined by patients, and therefore their expectations and experiences, but also their actual treatment decisions. Our findings also attend to the argument made by Spar (2006), by confirming that the commercialisation of add-ons requires careful regulation for protecting patients from the allure of the market. In other Western countries where treatment is largely funded publicly (for instance, in the Netherlands or France), add-ons are relatively uncommon.

In this article, we introduce the concept of “crafting hope” to underline how IVF patients navigate the complex dynamics and tensions that characterise the IVF trajectory in a highly competitive market. Our findings show a broad concern with making emotionally-driven treatment decisions in a stressful and uncertain context such as fertility care. We argue that patients crafted hope through careful consideration of whether to emotionally and financially invest in potentiality or curtail and temper hope to prevent potential harm. This dynamic of hope provided the foundation for patients to claim their agency and active role in treatment decisions and to refuse the notion that their vulnerability makes them uncritically accept any additional treatment available.

Given that numerous add-ons are available through an unregulated market, patients carry the burden of decisions about what to include in their treatment in a context that promotes the model of the persistent patient who wants to “try anything possible” to avoid future regret. As our findings show, not all patients adhere to this model; however, these narratives widen inequalities in fertility care. While for patients who are able and willing to pay for additional treatments, these can sustain the hope for a biological child, for those with less financial resources the availability of add-ons may hinder patients’ ability to avoid regret and find reproductive resolution. Delegating treatment decisions to IVF patients can increase their emotional and financial burden. Therefore, we argue that the dynamics presented in this article should be taken into consideration as a key aspect of the ethics of care in IVF, and clinics should consider carefully what kind of add-ons they are offering, to whom, and in what circumstances.

## CRediT author statement

Manuela Perrotta: Conceptualization, Methodology, Writing – original draft, Writing – review & editing, Supervision, Project administration, Funding acquisition. Josie Alice Hamper: Conceptualization, Methodology, Investigation, Formal analysis, Data curation, Writing – original draft, Writing – review & editing.
